# Proteomics Profiling of Neuron-Derived Small Extracellular Vesicles from Human Plasma: Enabling Single-Subject Analysis

**DOI:** 10.3390/ijms22062951

**Published:** 2021-03-14

**Authors:** Federica Anastasi, Silvia Maria Masciandaro, Renata Del Carratore, Maria Teresa Dell’Anno, Giovanni Signore, Alessandra Falleni, Liam A. McDonnell, Paolo Bongioanni

**Affiliations:** 1 National Enterprise for NanoScience and NanoTechnology, Scuola Normale Superiore, 56127 Pisa, Italy; federica.anastasi@sns.it; 2 Fondazione Pisana per la Scienza ONLUS, 56017 San Giuliano Terme, Italy; mt.dellanno@fpscience.it (M.T.D.); g.signore@fpscience.it (G.S.); 3 National Research Council, Institute of Clinical Physiology Research, 56124 Pisa, Italy; silvia.maria@virgilio.it; 4 Unit of Applied Biology and Genetics, Department of Clinical and Experimental Medicine, University of Pisa, 56126 Pisa, Italy; alessandra.falleni@unipi.it; 5 Severe Acquired Brain Injuries Dpt Section, Azienda Ospedaliero-Universitaria Pisana, 56126 Pisa, Italy; bongioanni.paolo@gmail.com; 6 NeuroCare Onlus, 56121 Pisa, Italy

**Keywords:** exosomes, small extracellular vesicles, neuron-derived vesicles, central nervous system, Parkinson’s disease, NES cells, proteomics, plasma, *L1CAM*, miR-155

## Abstract

Small extracellular vesicles have been intensively studied as a source of biomarkers in neurodegenerative disorders. The possibility to isolate neuron-derived small extracellular vesicles (NDsEV) from blood represents a potential window into brain pathological processes. To date, the absence of sensitive NDsEV isolation and full proteome characterization methods has meant their protein content has been underexplored, particularly for individual patients. Here, we report a rapid method based on an immunoplate covalently coated with mouse monoclonal anti-*L1CAM* antibody for the isolation and the proteome characterization of plasma-NDsEV from individual Parkinson’s disease (PD) patients. We isolated round-shaped vesicles with morphological characteristics consistent with exosomes. On average, 349 ± 38 protein groups were identified by liquid chromatography–tandem mass spectrometry (LC-MS/MS) analysis, 20 of which are annotated in the Human Protein Atlas as being highly expressed in the brain, and 213 were shared with a reference NDsEV dataset obtained from cultured human neurons. Moreover, this approach enabled the identification of 23 proteins belonging to the Parkinson disease KEGG pathway, as well as proteins previously reported as PD circulating biomarkers.

## 1. Introduction

Parkinson’s disease (PD) is a neurological disorder characterized by the progressive loss of nigral dopaminergic neurons and subsequent dopamine deficiency, which eventually results in motor symptoms and cognitive impairment [1,2]. PD early diagnosis is challenging because of the late onset of symptoms, therefore reliable biomarkers are needed for earlier treatment and disease monitoring [3]. Many circulating markers for PD have been reported: the level of α-synuclein (α-syn) in cerebrospinal fluid (CSF) was found to be lower in PD patients than in controls [4]; similarly, the levels of phosphorylated α-syn and total α-syn in patient plasma have been investigated for patient diagnosis [5]. Other proteins have been proposed, including Parkinson disease protein 7 and its isoforms in plasma [6], Apolipoprotein 1 in plasma [7], and Amyloid β1-42 and the tau protein in CSF [8]. However, it was reported that platelet contamination or variation in hemolysis could confound the reliable quantification of the blood Parkinson disease protein 7 and α-syn levels and thus further studies are needed to validate these proposed markers [9,10].

Small extracellular vesicles (sEV, diameter: 30–150 nm) are secreted by all cell types, are present in all body fluids, and carry molecules such as proteins, lipids, DNA, RNA, and miRNAs [11,12]. Their lipid bilayer of the outer membrane allows sEV to be stable for long periods of time and prevents degradation of their molecular content [13]. Moreover, sEV can cross the blood–brain barrier and therefore represent an easily accessible source of central nervous system (CNS) biomarkers [14]. In the CNS, sEV are involved in intercellular communication, neuronal plasticity, and myelin regeneration in both physiological and pathological states [15,16,17]. sEV-based biomarker detection is rapidly gaining interest within the neurodegenerative disease research communities [18]. In fact, it has been demonstrated that sEV transport pathogenic proteins in PD [19], and their molecular content has been shown to correlate with PD onset and progression [20]. sEV of neuronal origin can be isolated using neuronal antigens, such as neural cell adhesion molecule L1 (*L1CAM*) [21]. The development of *L1CAM* immuno-precipitation protocols for the isolation of neuron-derived sEV (NDsEV) was pioneered by the groups of Goetzl at the University of California and Zhang at the University of Washington [22,23]. The protocols involve the use of biotinylated anti-*L1CAM* antibodies and a streptavidin resin, or the prior immobilization of the anti-*L1CAM* antibody onto M-270 epoxy beads followed by incubation with the sample. These *L1CAM* workflows have demonstrated that NDsEV represent a source of biomarkers, in which ELISA, Western blot, or targeted proteomics analysis has been used to follow the level of specific target proteins (α-syn, tau protein, DJ-1, and Aβ42) [3,24,25,26].

NDsEV provide new opportunities to identify markers of neuropathological conditions and may indicate how NDsEV contribute to disease progression. However, the low levels of NDsEV in peripheral biofluids and the low total amount of protein within sEV constitute a formidable analytical challenge, requiring high sensitivity NDsEV isolation, proteomics sample preparation, and LC-MS/MS methods. To date there is an evident lack of proteomics investigations of NDsEV from individual patients, which is essential for the identification of novel markers and the development of personalized profiling for disease diagnosis and prognosis. Jiang and colleagues recently performed a longitudinal study of neuronal sEV isolated by *L1CAM* from serum and found that the combination of α-syn and clusterin levels differentiated PD from other proteinopathies and from multiple system atrophy. The same group has reported the identification of 512 proteins using NDsEV pooled from multiple patients [27]. The ability to characterize the NDsEV proteome from individual patients is a necessary step for the identification of new biomarkers, and to explore the full potential of NDsEV for personalized medicine. In light of these considerations, here we report the development of a rapid and cost-effective procedure for the isolation of NDsEV from the plasma of individual patients, followed by their comprehensive proteomics analysis. The morphological and proteomics characterization of the isolated NDsEV confirmed the efficient isolation of vesicles consistent with exosomes.

To investigate the specificity of the NDsEV isolation method, we compared the protein profiles obtained from plasma NDsEV with those obtained from sEV isolated from the supernatant of cultured human neurons established in vitro from the differentiation of human neuroepithelial stem (NES) cells. NES cells have been derived from several anatomical areas of the developing neural tube [28,29,30], and evidence demonstrates that they are highly expandable without undergoing immortalization processes and retain in vitro a remarkable neurogenic potential, thus allowing the generation of a reliable platform of human neurons [29,30]. Since the NES-cell-derived neurons were not obtained through reprogramming techniques, they preserve the transcriptional and epigenetic profile of their corresponding counterpart in vivo [29]; for this reason, the sEV collected from this platform represent an optimal reference for the comparison with NDsEV isolated from patient plasma to gauge NDsEV specificity.

The isolation of NDsEV from plasma and the proteomics analysis enabled the identification of many proteins shared with the NES-cell reference database; these are also annotated as having elevated neural expression in the Human Brain Proteome database. This included 23 proteins belonging to the PD KEGG pathway and several circulating biomarkers. Note: In this study the term sEV is used instead of exosome because, according to guidelines published by the International Society of Extracellular Vesicles, the term exosome should only be used if it can be explicitly demonstrated that they have a late endosomal origin [31,32].

## 2. Results

### 2.1. Proteomics of Small Extracellular Vesicles from Cultured Human Neurons

We first sought to create a reference database of small extracellular vesicles (sEV) proteins from cultured neurons with which the proteins from plasma neuron-derived small extracellular vesicles (NDsEV) could be compared. Neurons obtained from neuroepithelial stem (NES) cells were differentiated to complete maturation as assessed by the expression of pan-neuronal markers (Figure S1) and media collected at the end of the differentiation process (see details in the Materials and Methods).

**Figure 1:**
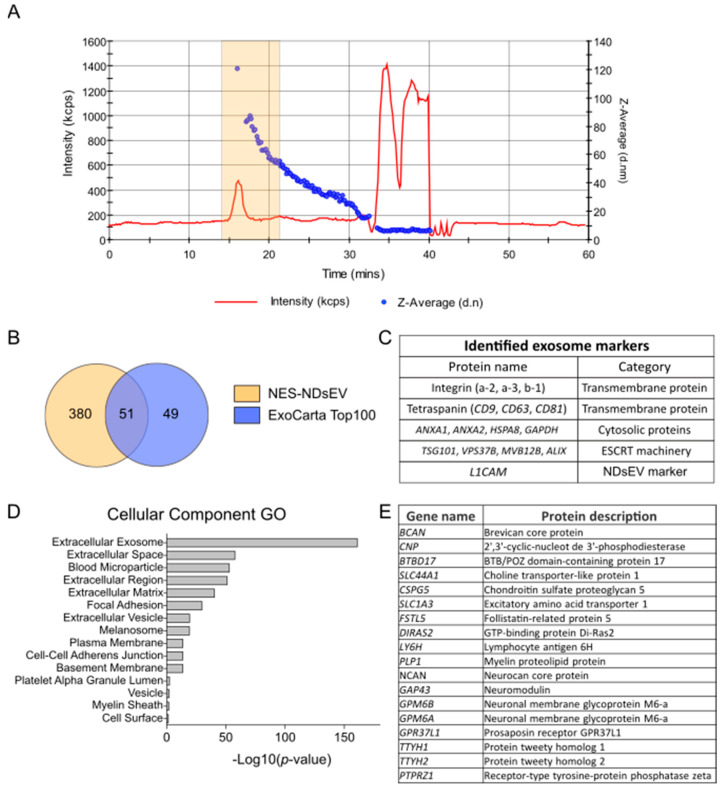
Cultured human neuron-derived small extracellular vesicles (NDsEV) isolation and characterization. (**A**) Size Exclusion Chromatography using in-flow Dynamic Light Scattering detection; the graph presents vesicle diameter (d.nm) in blue and the derived count rate in red. The orange box highlight indicates the fractions selected for NDsEV isolation. (**B**) Venn diagram of the proteins identified in the neuroepithelial stem cell neuron-derived sEV (NES-NDsEV) and their overlap with the ExoCarta Top 100 database. (**C**) Exosome markers identified in the NES-NDsEV. (**D**) Cellular component Gene Ontology enrichment analysis of the NES-NDsEV proteins using the DAVID database [33]. (**E**) Identified proteins in NES-NDsEV in common with the Human Brain Proteome (Human Proteome Atlas) database annotated as significantly enriched genes in the brain (488 entries).

Neuron-derived small extracellular vesicles from NES-cell-derived neurons (NES-NDsEV) were isolated by Size Exclusion Chromatography (SEC) and sEV proteins analyzed by liquid chromatography–tandem mass spectrometry (LC-MS/MS). A preliminary SEC-HPLC analysis with in-flow dynamic light scattering was performed to characterize the elution profile in terms of particle size and scattering intensity. On the basis of the vesicles’ elution profile (Figure 1A) and of the protein elution profile (Figure S2), it was determined that sEV eluted in the fractions from 14 to 22 min, which reported an extracted average diameter of 70.5 nm.

The NES-NDsEV proteomics analysis using the S-trap protocol led to the identification of 431 protein groups from a total of 3.1 μg of digested peptides (Supplementary Dataset-SD1). The identified proteins included 51 of the exosomal proteins listed in the ExoCarta Top 100 database (Figure 1B) as well as the ESCRT machinery proteins *TSG101*, *VPS37B*, *ALIX*, and *MVB12B*, which are characteristic of their endosomal origin [27]. The proteins identified from the NES-NDsEV included neural adhesion molecule L1 (*L1CAM*), which was used here to isolate NDsEV from patient plasma (Figure 1C).

A Gene Ontology (GO) analysis of the NES-NDsEV proteins showed that “extracellular exosome” was the most enriched cellular component term, with 311 counts and an FDR of 1.4 × 10^−161^ (Figure 1D, Table S1). We then investigated whether the NES-NDsEV retained neuronal cell specific markers, by comparing the NES-NDsEV proteins with the Human Brain Proteome database of the Human Protein Atlas [34]. This database (download: December 2020) reports 16,227 genes that have been detected in the brain, 2587 genes annotated as having elevated expression in the brain, 488 genes significantly enriched in the brain, and 33 genes that are exclusive to the brain. The 431 protein groups identified from NES-NDsEV included 57 proteins reported to having elevated expression in the brain (Table S2) and 18 that are significantly enriched in the brain (Figure 1E). Among these 18 proteins, we highlight two neuronal membrane glycoproteins (*GPM6A* and *GPM6B*) and myelin glycolipid protein (*PLP1*), all known to be involved in neurite growth [35].

### 2.2. Isolation and Morphological Characterization of Plasma NDsEV from PD Patients

**Figure 2:**
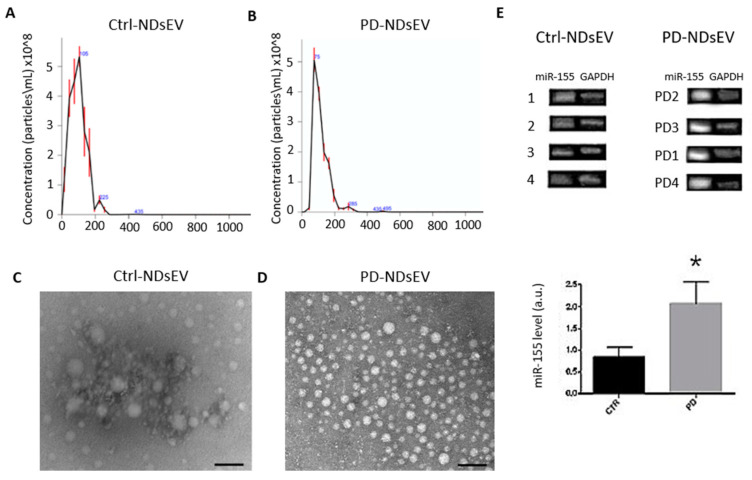
Plasma neuron derived small extracellular vesicles (NDsEV) characterization. (**A**) Nanoparticle-tracking analysis of plasma-NDsEV isolated from healthy control plasma samples and (**B**) Parkinson disease patient plasma. (**C**) Representative TEM images of plasma-NDsEV from healthy controls and (**D**) PD patients. Scale bars: 100 nm. (**E**) miR155 expression in 4 PD patients’ NDsEV and 4 healthy controls’ NDsEV; the histograms report the mean of the densitometric values of the bands of controls (in black) and of PD (in grey) normalized with respect to the *GAPDH* ± SD * *p* < 0.05.

We developed a workflow for the isolation and the proteomics characterization of NDsEV from the plasma of individual patients. Blood was collected from four PD patients and four healthy controls, and the NDsEV isolation performed using platelet-free plasma, as recommended by the International Society of Extracellular Vesicles guidelines, MISEV 2018 [31]. An immunoplate covalently coated with antibodies for *L1CAM* was used for the isolation of NDsEV; *L1CAM* is a characteristic marker of NDsEV and has been previously used for their immunoprecipitation [21]. Of note, *L1CAM* was also identified in the NES-NDsEV (see above). NDsEV isolated from the plasma of PD and the age-matched control subjects were characterized using nanoparticle-tracking analysis (NTA) and transmission electron microscopy (TEM). NTA revealed the presence of vesicles with an average diameter of 97 ± 49 (mean ± SD) and 115 ± 49 (mean ± SD) for Control-NDsEV and PD-NDsEV, respectively, consistent with exosome size [36] (Figure 2A,B). The TEM analysis revealed that the *L1CAM*-immunoplate procedure isolated round-shaped intact vesicles for both PD (Figure 2C) and control subjects (Figure 2D).

As an additional control, we determined the level of miR-155 in the PD and control subjects. miR-155 is a regulator of α-synuclein (α-syn) and induced an inflammatory response in PD [37], whose presence in exosomes was previously demonstrated [38]. We confirmed that NDsEV isolated with the *L1CAM*-immunoplate from PD patient plasma contained higher levels of miR-155 than those isolated from age-matched healthy individuals (E).

### 2.3. Proteomics Analysis of Plasma-NDsEV from PD Patients

**Figure 3:**
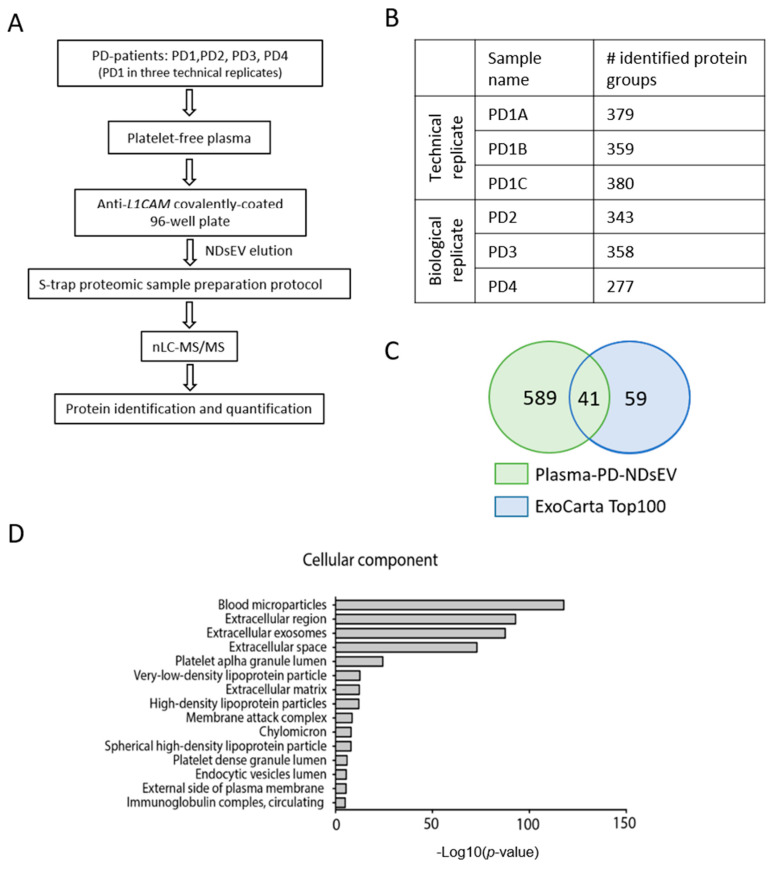
(**A**) NDsEV proteomics workflow: NDsEV were isolated from platelet-free plasma by *L1CAM* immune-precipitation, and S-traps used to digest the proteins and to purify the peptides that are then analyzed by LC-MS/MS for protein identification and quantification. (**B**) Number of protein groups identified in each plasma sample. (**C**) Venn diagram of the proteins identified in the replicates and the ExoCarta Top 100 database. (**D**) Cellular component Gene Ontology enrichment analysis of the proteins identified from plasma-NDsEV of PD patients using the DAVID database [33].

The plasma of four PD patients was collected for NDsEV isolation and further proteome profiling: this study was designed to assess the technical reproducibility and biological variability. After the isolation of *L1CAM*-positive NDsEV, the proteins were extracted, digested, and identified by LC-MS/MS (Figure 3A). Using just 150 μL of plasma from each patient, an average of 349 ± 38 protein groups were identified from the NDsEV (Figure 3B, Supplementary Dataset SD2), 41 of which are contained in the Top 100 exosome proteins annotated in the ExoCarta database (Figure 3C, Table S3). The exosome proteins *ANXA2*, *GAPDH*, *ALDOA*, and *A2M* were identified in the NDsEV of all PD patients. Other common exosome proteins were identified in the PD-NDsEV samples but not in all patients (e.g., *CD9*, *ANXA1*, *ITGB1*, and *HSPA8*).

In total, 177 protein groups were identified in all patient plasma-NDsEV samples (Supplementary Dataset–SD3); the GO analysis of this protein list highlighted the presence of blood microparticles (count: 74, FDR: 1.5 × 10^−112^), extracellular region proteins (count: 127, FDR: 1.9 × 10^−99^), and extracellular exosomes (count: 140, FDR: 7.9 × 10^−88^) as the top three enriched cellular component categories (Figure 3D). The full list of the “Cellular Component” GO terms with relative counts and FDR-corrected *p*-value is available in Table S4. When the GO analysis was repeated using all proteins identified from NDsEV, i.e., also including those detected in a subset of patients, “extracellular exosomes” was the most enriched term (counts: 411, FDR: 1.2 × 10^−198^, Table S5).

Extracellular exosomes are defined as sEV that originate from late endosomes [39]; in this study the “extracellular exosome” category was strongly enriched. We thus investigated the presence of the endosomal sorting complexes required for transport (ESCRT) machinery and the presence of proteins associated with endosomes. Here, the ESCRT machinery proteins (e.g., *TSG101*, *MVB12*, *VPS*, and *ALIX*) [40] were not identified, presumably due to the high complexity of the plasma samples and the very small protein amount available that prevented the identification of the less abundant proteins. Nevertheless, in the PD-NDsEV, we identified 20 proteins annotated as belonging to the “early endosome” GO cellular component category (GO: 0005769, Table S6) and 44 proteins associated with “endosome” (GO: 0005768, Table S7); the latter included Early endosome antigen 1 (*EEA1*), Syntaxyn-7 (*STX7*), and Lysosome-associated membrane glycoprotein 1 (*LAMP1*). These results indicate the successful enrichment of vesicles, consistent with exosome characteristics.

**Figure 4:**
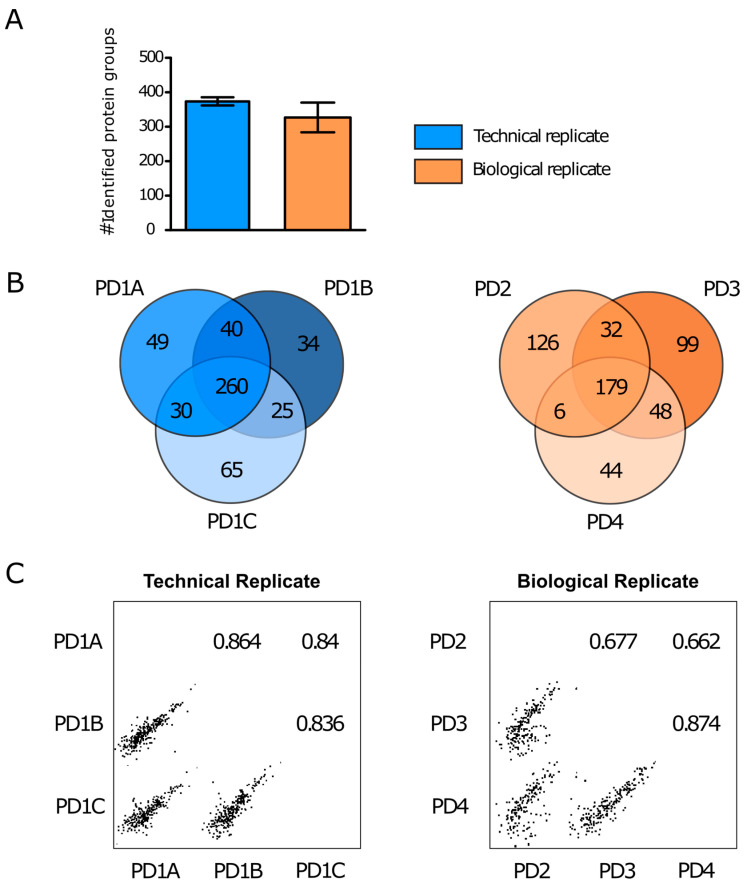
(**A**) Average number of identified proteins groups of PD-NDsEV and relative standard deviations for the technical replicates (PD1A, PD1B and PD1C) and biological replicates (PD2, PD3 and PD4). (**B**) Venn diagram showing the number of common and unique proteins among the technical replicates and among biological replicates of PD-NDsEV. (**C**) Protein intensity correlation among the technical replicates and among biological replicates; the figures report the Pearson’s correlation coefficient extracted with Perseus software [41].

The average number of protein groups identified from the PD-NDsEV was 373 and 326 for the technical and biological replicates, respectively (Figure 4A). Venn diagrams show that 260 and 179 protein groups were identified in all technical and biological replicates, respectively (Figure 4B). The variability in the number of identified proteins and detected protein levels (Figure 4C) was lower for the technical replicates than for the biological replicates. A comparison of the proteins from PD-NDsEV and the Human Brain Proteome resulted in two proteins that are highly enriched in the brain, namely, Tubulin beta-2A chain (*TUBB2A*), which is involved in neuron migration, and Myelin transcription factor 1-like protein (*MYT1L*), which is involved in neuronal differentiation. The PD-NDsEV contained an additional 18 proteins that are reported as having elevated expression in the brain (Table 1). A comparison of the PD-NDsEV proteins with those obtained from NES-NDsEV revealed 213 protein groups in common (Supplementary Dataset SD4), 93 of which were identified in all PD-NDsEV (Supplementary Dataset SD5).

### 2.4. Potential Plasma-NDsEV Markers of PD

The experiments reported here were performed using initial aliquots of 250 μL of platelet-free plasma, of which 150 μL was consumed for each experiment (see Methods for detail). Despite the low amount of plasma, the procedure allowed the identification of proteins previously reported to be involved in the pathogenesis of PD. From the PD patients’ plasma-NDsEV, we identified and quantified 23 proteins (Table 2) that belong to the PD KEGG pathway (HSA05012, 249 entries) [42,43].

Of particular interest is the presence of 10 proteins of the 20S Proteasome complex (*PSMA1-3*, *PSMA5-7*, *PSMB1*, *PSMB3*, and *PSMB5-6*), which is a part of the ubiquitin proteasome system (UPS), known to be impaired in PD and the major cause of neuronal degeneration [44]. Among these 23 proteins, we identified Parkinson’s disease protein 7 (*PARK7*), which was previously proposed as a candidate biomarker after demonstrating increased levels in the plasma-NDsEV of PD patients with respect to the controls [25]. Moreover, the NDsEV proteins identified from the PD patient plasma samples also included proteins previously annotated as circulating markers of PD (e.g., Gelsolin, Amyloid P component, Clusterin, and Stromal cell-derived factor 1). Gelsolin was reported to be progressively upregulated from mild PD to severe PD [45]; the Amyloid P component behaves as an acute-phase reactant and was proposed as a PD marker [46]. Clusterin has been repeatedly linked to PD, and its plasma levels have been found to be increased in PD patients [47]. Stromal cell-derived factor 1 (*CXCL12*) was confirmed to be positively correlated with α-syn in post-mortem brains tissues of PD patients and increased in the blood of PD patients [27]; moreover, in the same study, α-syn increased the production of *CXCL12* in microglia [48].

## 3. Discussion

The isolation and characterization of NDsEV has gained increasing interest owing to their potential to provide diagnostic and prognostic markers of different CNS pathologies [21,27,49]. Here, we reported a workflow for the characterization of plasma-sEV of neuronal origin. For the first time, a 96-well plate covalently pre-coated with the anti-neuronal adhesion molecule L1 (*L1CAM*) was used for NDsEV proteome profiling; this has significant advantages with respect to the available approaches, in terms of lower cost, increased ease of use, automatization of NDsEV enrichment, and the low plasma volumes needed. The morphological characterization of the sEV by NTA and TEM showed that the procedure leads to the isolation of round-shaped vesicles with exosome characteristics. Of note, we found that the NDsEV of PD patients exhibited increased expression of miR-155. miR-155 has a central role in the inflammatory response caused by α-synuclein aggregates [37], and it is known that miR-155 is transported into extracellular vesicles [38,50]; no comparison of miR-155 levels in PD plasma-NDsEV has been previously reported.

The major aim of this proof-of-concept study was the development of a low-cost and high-throughput methodology for NDsEV isolation for profiling the NDsEV proteome of individual patient plasma samples. NDsEV isolated by the immunoplate were processed for LC-MS/MS-based proteomics using S-traps because they enable the sensitive and efficient analysis of volume-limited samples [51]. The combination of immunoplate isolation of NDsEV and S-trap based proteomics sample preparation provided the performance necessary for the characterization of the NDsEV proteome from individual patients. An average of 349 NDsEV protein groups were identified from each PD patient using only 150 µL of plasma. The successful enrichment of vesicles with exosome characteristics was confirmed by the identification of exosome markers (*ANXA2*, *ITGB1*, and *CD9*), markers of the endosomal biogenesis pathway (Early endosome antigen 1, *EEA1*), and finally by a GO analysis that demonstrated an enrichment of the “extracellular exosomes” term.

A reference protein dataset was obtained using human neuron-derived sEV generated upon in vitro differentiation of NES cells. A comparison of the proteins identified from the NES-cell-derived neurons (NES-NDsEV) with the Brain Atlas database led to the identification of 18 proteins that are annotated as highly expressed in the brain. These proteins included the *L1CAM* protein used here to isolate plasma-NDsEV. A comparison of the proteins identified from the NES-NDsEV and the plasma-NDsEV resulted in 213 protein groups that were present in both datasets, again indicating that the *L1CAM*-NDsEV approach efficiently isolated NDsEV. It should be noted that the NES-derived neurons were cultured in the absence of serum in the culture media. Accordingly, the proteins found in both the plasma-NDsEV and NES-NDsEV samples can only originate from NDsEV and not from blood contamination of the plasma-NDsEV preparations.

### Concluding Remarks

Here, we describe an efficient method for the comprehensive proteome profiling of NDsEV isolated from the plasma of individual patients. The procedure provides the capabilities to investigate NDsEV as a source of easily accessible biomarkers and to obtain indicative information about PD progression. The methodology here described proved to be reliable and cost-effective and, more importantly, it can be easily applied to all CNS disorders, for diagnostic biomarkers identification or longitudinal follow-up and monitoring of patient response.

## 4. Materials and Methods

### 4.1. Maintenance and Differentiation of the Neuroepithelial Stem Cells

Human neocortical neurons were differentiated from neuroepithelial stem cells (NES) derived as previously described [29,30]. NES cells were kept in proliferation in T25 flasks coated with poly-L ornithine (0.01%), laminin (5 μg/mL), and fibronectin. NES medium, used to keep cells in proliferation, was composed as follows: DMEM/F12 (Gibco) with the addition of B27 supplement (1:1000, Invitrogen), N2 supplement (1:100, Gibco), 20 ng/mL FGF2 (Gibco), 20 ng/mL EGF (Gibco), 1.6 mg/mL glucose, 20 μg/mL insulin (Sigma), and 5 ng/mL BDNF. Cells were split 1:2 with 0.25% trypsin and half volume of the medium was changed every 2–3 days. Neuronal differentiation of NES cells was performed in two steps. For the pre-differentiation step, NES cells were seeded at a density of 0.5 × 10^5^ cells/cm^2^ in a T25 coated flask in NES medium without EGF and FGF2. After seven days, the cells were dissociated and re-plated at a density of 0.8–1 × 10^5^ cells/cm^2^ in differentiation medium composed of DMEM/F12 and Neurobasal medium (1:1 ratio) supplemented with B27 (2%), N2 (1%), and BDNF (20 ng/mL). Half of the medium was changed every 2–3 days and neurons were differentiated for up to three months.

### 4.2. Small Extracellular Vesicles Enrichment from Human Neurons Culture Media

The media were collected from cultured neocortical neurons, pooled, and filtered using a 0.2 μm filter to remove cell debris and larger vesicles, and then stored at −80 °C. All samples underwent one freeze–thaw cycle only. After thawing, the media was concentrated using a Pierce Protein Concentrator (100 K MWCO, 2–6 mL, Thermo Fisher Scientific) to a final volume of 200 μL, prior to sEV purification by Size Exclusion Chromatography (SEC), as follows. A Superose 6 Increase column (10/300, GE Healthcare) was coupled to a Dionex Ultimate 3000 HPLC (Thermo Fisher Scientific) equipped with a photodiode array detector, a thermostated autosampler/fraction collector maintained at 5 °C, and interfaceable with a Zetasizer Dynamic Light Scattering (DLS, Malvern) suitable for flow measurements. Sample separation was performed using a 50-min run with filtered phosphate buffer saline (PBS) as elution buffer. Typically, 200 μL were injected in each run. Molecular weight calibration of the SEC column (Figure S3) was performed using high molecular weight protein standards (Phenomenex). The calibration mixture contained thyroglobulin, bovine serum albumin, and myoglobin. Blue dextran 2000 (Cytiva) was used to determine the void volume. A preliminary run using the DLS in line with the SEC-HPLC system was used to determine the elution profile of the extracellular vesicles and proteins contained in a sample matrix (Figure S2). Fractions containing NDsEV without protein contamination were selected based on the following criteria: (1) high scattering counts; (2) measured diameter >50 nm; and (3) no absorption measured at 280 nm, deriving from the lower molecular weight components. The fractions of interest were pooled and concentrated using a 3 KDa Protein Concentrators (Pierce 3 MWCO, 0.5 mL, Thermo Fisher Scientifics). The resulting suspension was then stored overnight at −20 °C prior to proteomics sample preparation.

### 4.3. Human Subject and Clinical Plasma Sample Collection

Blood was withdrawn from four PD patients and four age-matched healthy subjects (age range between 50 and 80), who did not suffer from any other neurological (cerebrovascular or neuroinflammatory/immune) diseases, severe brain injuries, and/or severe non-neurological illnesses (cardiovascular and blood diseases, kidney, liver or pancreas failure, or immune disorder). The blood was collected into EDTA-treated tubes. Plasma was obtained by centrifugation at 150× *g* for 15 min at room temperature, followed by centrifugation at 9600× *g* to obtain platelet-free plasma, which was then aliquoted and stored at −80 °C. All plasma aliquots underwent a single freeze–thaw cycle only.

### 4.4. NDsEV Enrichment from Plasma

To remove cell debris and larger vesicles, the thawed plasma samples of 250 µL underwent three centrifugation steps: 10 min at 300× *g*, 20 min at 1200× *g*, and 30 min at 10,000× *g*. The plasma was then diluted 1:1 with PBS. In this study we used a 96-multiwell ELISA plate covalently pre-coated with the anti-neuronal adhesion molecule L1 (*L1CAM* or CD171) (HansaBioMed Life Sciences-Lonza), using the clone UJ127 previously accepted for blood-NDsEV isolation [24,52]. Three wells were used for each sample and 100 µL of 1:1 plasma:PBS were added to each well. The plate was kept under shaking for 20 min at RT and incubated overnight at 4 °C. Four washing steps were performed using PBS containing 0.05% Tween 20. NDsEV were then collected using 15 µL of elution buffer (beads elution buffer, HansaBioMed Life Sciences-Lonza) and 25 µL of PBS added into each well. The NDsEV suspensions were then stored at −20 °C.

### 4.5. Particle Size-Distribution

NDsEV size distribution was determined by nanoparticle-tracking analysis (NTA) using a NanoSight NS300 (Malvern Panalytical). The NDsEV suspension was diluted 1:20 with PBS prior to analysis. The scattered light from the individual particles was recorded and the particle trajectory and displacement analyzed by the software NTA 3.3; particle size distribution was determined from the observed Brownian motion according to the Stokes–Einstein equation. NTA analysis was individually performed on the sEV obtained from the plasma of healthy and Parkinsonian patients.

### 4.6. Transmission Electron Microscopy

Transmission electron microscopy (TEM) was performed to characterize the morphology of NDsEV isolated from the plasma of PD patients and age-matched healthy controls. Samples were prepared using a negative staining procedure; 10 μL of sample was placed onto 200-mesh formvar/carbon-coated copper grids and allowed to settle for 3 min at RT. The grids were washed with distilled water and 20 μL of an aqueous solution of uranyl acetate (2% *w*/*v*), then applied for 30 s. The samples were then analyzed with a Jeol 100 SX (Japan) transmission electron microscope operating at 80 KV. Micrographs at 25,000–40,000 × direct magnification were obtained with an AMTXR80b Camera System.

### 4.7. RNA Extraction and RT-PCR

RNA was extracted using the miRNeasy Mini KIT (Qiagen, cat. no. 217004) according to the protocol provided by the manufacturer. Briefly, 200 μL of PBS and 3 pmols of cel-miR-39 RNA were added to all samples as a spike. sEV samples were lysed with 1 mL of QIAzolLS and 200 μL of chloroform. After centrifugation, the aqueous phase was collected, filtered, and the RNA eluted in 40 μL of nuclease-free water. RNA quantification was performed using a NanoDrop spectrophotometer (Thermo Fisher Scientific). Starting from 1 μg of total RNA from each sample, cDNA was retro-transcribed using the miScript PCR Starter Kit (QIAGEN), according to the following reaction: 4 μL of HiSpec Buffer 5×, 2 μL of the nucleic mix, 2 μL of Reverse Transcriptase, and 11 μL of RNA template, for a final volume of 20 μL. The reaction was performed with two incubation steps (1 h at 37 ° C and 5 min at 95 °C). miRNA 155 expression was assayed in Control-NDsEV and PD-NDsEV, and their levels normalized relative to *GAPDH*. miR-155 was amplified using 5p TTAATGCTAATCGTGATAGGGGT as the forward primer, and the UP1 included in the kit was used as a reverse primer. *GAPDH* was amplified with the following primers: Fw (5′-GCCTTCCGTGTTCCTACCC-3′), Rev (5′-TGCCTGCTT CACCACCTTC-3′). cDNA was amplified by sqPCR using GoTaq Green Master Mix according to the following program: initial denaturation at 93 °C for 3 min, followed by 35 cycles of denaturation at 93 °C (30 s), annealing at 65 °C for miR-155 and 62 °C for *GAPDH*, extension at 72 °C (30 s), and the last final extension at 72 °C for 10 min.

### 4.8. Proteomics Sample Preparation

A lysis buffer consisting of 10% sodium dodecyl sulfate (SDS), 100 mM triethylammonium bicarbonate (TEAB), pH 7.55, was added to the NDsEV suspensions in a ratio of 1:1 (*v*/*v*). The vesicles were then lysed by sonication using a Bioruptor Pico (Diagenode, Seraing, Belgium; 20 cycles of 30 s ON and 30 s OFF, 4 °C). Proteins were denatured by incubation for 5 min at 95 °C, reduced using 20 mM dithiothreitol (30 min incubation at 45°C), and then alkylated using 40 mM iodoacetamide (30 min incubation at room temperature in the dark). Proteolytic digestion was performed using S-trap Micro filters (ProtiFi, Huntington, NY) according to manufacturer’s instructions with slight modifications [49]. Briefly, the protein mixture was acidified with 12% aqueous phosphoric acid using an acid:sample ratio of 1:10. The samples were diluted six times with a protein-binding buffer (90% methanol, 100 mM TEAB, pH 7.1) and then loaded on to the S-trap filters by centrifugation at 4000× *g*. The loaded proteins were then rinsed 9 times with 150 μL of the protein-binding buffer. Proteolytic digestion was performed by the addition of 25 μL of digestion buffer (0.3 μg Trypsin-LysC, 50 mM TEAB, pH 8) and incubated at 37 °C for 18 h. Trypsin/Lys-C mix Mass Spec grade was purchased from Promega (Madison, WI, USA). Proteolytic peptides were eluted with 40 μL each of 50 mM TEAB and 0.2% formic acid. Hydrophobic peptides were eluted with 35 μL of 50% acetonitrile containing 0.2% formic acid. The three eluates were pooled together, dried, and suspended in 11 µL of LC-MS water. The total peptide concentration was quantified using the Pierce™ Quantitative Colorimetric Peptide Assay (Thermo Fisher Scientific, Rockford, IL, USA), using 1 μL of the peptide solution. The peptides were then stored at −20 °C in 0.5 mL LoBind tubes (Eppendorf, Hamburg, DE).

### 4.9. Mass Spectrometry Analysis

The samples were diluted 1:1 with 10% formic acid and injected into an Easy-nLC 1000 coupled to an Orbitrap Fusion mass spectrometer (both Thermo Fisher Scientific, Bremen, Germany). Peptides were separated in reverse phase mode online using an EASY-Spray PepMap^TM^ analytical column (ES803: 50 cm × 75 µm, C18, 2 µm, 100 Å; Thermo Scientific) equipped with an Acclaim PepMap^TM^ trap-column (2 cm × 75, C18, 3 μm, 100 Å; Thermo Scientific). A quality control experiment was performed after every analysis using a peptide mixture from bovine serum albumin (Sigma-Aldrich; St. Louis, MO, USA) to assess carry-over and to test overall chromatographic performance. Then 1.5 µg of digested peptides were separated using a 145 min gradient and a flow rate of 300 nL/min. Buffer A consisted of LC-MS grade water with 0.1% formic acid and Buffer B of LC-MS grade acetonitrile with 0.1% formic acid. Peptides were loaded at 800 bar and separated by a non-linear 145 min gradient: 0–1 min, 5% B; t = 104 min, 5–22% B; t = 120 min, 22–32% B, t = 130 min, 32–90% B; t = 145 min, 90% B. The Orbitrap Fusion mass spectrometer operated in positive ion mode using a data-dependent Top Speed mode with a 3 s cycle time. The nESI voltage was 2100–2300 V and the ion transfer tube temperature was 275 °C. The MS method consisted of a full mass spectrum in the Orbitrap (scan range: 375 to 1500 *m*/*z*, 120 K resolution, AGC target of 4 × 10^5^, max injection time of 50 ms). Monoisotopic precursor selection and a dynamic exclusion of 60 s were used. Ions with charge states from 2+ to 7+ and an intensity greater than 5 × 10^3^ were selected for HCD fragmentation at 30% NCE using an isolation window of 1.6 *m*/*z*. MS/MS acquisition were performed in the linear ion trap with a rapid scan rate, AGC target of 2 × 10^3^, and 300 ms maximum injection time.

### 4.10. Protein Identification

Proteome Discoverer 2.1 (Thermo Scientific) was used to process the LC-MS/MS raw data, using the SequestHT search engine [53] and the UniProt *Homo sapiens* protein database (April 2019, 20.428 entries) supplemented with a home-made common contaminants database (250 sequences). The MS/MS spectra were searched using the following parameters: 10 ppm precursor mass tolerance and 0.6 Da fragment mass tolerance; up to 2 missed cleavages; minimum peptide length 7 amino acids; methionine oxidation (+15.995 Da) and acetylation (+42.01 Da, N termini) as dynamic modifications; and cysteine carbamidomethylation (+57.021 Da) as fixed modification. The search engine results were then filtered for a 1% false discovery rate (FDR) using the Percolator algorithm [54] and filtered for a minimum peptide XCorr score of 1.8. At least one unique peptide was required for definitive protein identification.

### 4.11. Data Analysis

The identified protein groups were compared using Venn diagrams (http://bioinformatics.psb.ugent.be/webtools/Venn/ (accessed: December 2020)). Gene ontology (GO) analysis was performed using the Database for Annotation, Visualization and Integrated Discovery (DAVID) v6.8 [33], with the whole *Homo sapiens* genome as statistical background. GO analysis was performed using a Fisher’s exact test followed by FDR multiple testing correction. The identified protein groups were compared with the EV markers present in the ExoCarta Top 100 database [55,56], with the Human Brain Proteome [57] and with the Parkinson Disease KEGG Pathway [43]. These comparisons were performed by string search of the protein names. Protein datasets were compared using the Pearson correlation coefficient to assess reproducibility. All proteomics data were analyzed using Perseus 1.5 software [41], Microsoft Excel, GraphPad Prism v5 for Windows (GraphPad Software, La Jolla California USA, www.graphpad.com). For quantitative analysis, common contaminants were removed prior to data normalization, and the raw protein intensities were then log2 transformed and median normalized.

## Figures and Tables

**Table 1:** The comparison of the identified proteins in plasma-NDsEV from PD patients with the Human Brain Proteome (Human Proteome Atlas) database led to the identification of two proteins (in grey) annotated as significantly enriched genes in the brain (488 entries) and 18 proteins annotated as elevated in the brain (2582 entries).

Uniprot ID	Gene Name	Description
Q13885	*TUBB2A*	Tubulin β-2A chain
Q9UL68	*MYT1L*	Myelin transcription factor 1-like protein
P09972	*ALDOC*	Fructose-bisphosphate aldolase C
P09172	*DBH*	Dopamine beta-hydroxylase
P0DP57	*SLURP2*	Secreted Ly-6/uPAR domain-containing protein 2
P11277	*SPTB*	Spectrin beta chain, erythrocytic
P60763	*RAC3*	Ras-related C3 botulinum toxin substrate 3
P12259	*F5*	Coagulation factor V
Q96KN2	*CNDP1*	Beta-Ala-His dipeptidase
Q9Y2T3	*GDA*	Guanine deaminase
P04180	*LCAT*	Phosphatidylcholine-sterol acyltransferase
P22792	*CPN2*	Carboxypeptidase N subunit 2
P32004	*L1CAM*	Neural cell adhesion molecule L1
P02766	*TTR*	Transthyretin
P04196	*HRG*	Histidine-rich glycoprotein
P16150	*SPN*	Leukosialin
Q93050	*ATP6V0A1*	V-type proton ATPase 116 kDa subunit a
P80723	*BASP1*	Brain acid soluble protein 1
P11171	*EPB41*	Protein 4.1
P0DP25	*CALM3*	Calmodulin-3

**Table 2:** Proteins of the PD KEGG pathway that were identified in the plasma-NDsEV from PD patients.

Uniprot ID	Gene Name	Description
P25705	*ATP5F1A*	ATP synthase subunit alpha, mitochondrial
P06576	*ATP5F1B*	ATP synthase subunit beta, mitochondrial
P0DP25	*CALM3*	Calmodulin-3
Q9NZT1	*CALML5*	Calmodulin like 5
P99999	*CYCS*	Cytochrome c
P04899	*GNAI2*	Guanine nucleotide-binding protein G i) subunit alpha-2
P11021	*HSPA5*	Endoplasmic reticulum chaperone BiP
Q99497	*PARK7*	Parkinsonism associated deglycase
P25786	*PSMA1*	Proteasome subunit alpha 1
P25787	*PSMA2*	Proteasome subunit alpha 2
P25788	*PSMA3*	Proteasome subunit alpha 3
P28066	*PSMA5*	Proteasome subunit alpha 5
P60900	*PSMA6*	Proteasome subunit alpha 6
O14818	*PSMA7*	Proteasome subunit alpha 7
P20618	*PSMB1*	Proteasome subunit beta 1
P49720	*PSMB3*	Proteasome subunit beta 3
P28074	*PSMB5*	Proteasome subunit beta 5
P28072	*PSMB6*	Proteasome subunit beta 6
P62979	*RPS27A*	Ubiquitin 40-S ribosomal protein S27a
P12235	*SLC25A4*	ADP/ATP translocase 1
Q13885	*TUBB2A*	Tubulin beta 2A class IIa
P68371	*TUBB4B*	Tubulin beta 4B class IVb
P10599	*TXN*	Thioredoxin

## Data Availability

The PD-NDsEV mass spectrometry proteomics data have been deposited to the ProteomeXchange Consortium (http://proteomecentral.proteomexchange.org) via the PRIDE partner repository [58] with the dataset identifier PXD020794. All relevant data supporting the findings of this study is available within this Manuscript and Supplementary Files. Any further question or request should be made to the corresponding authors.

## Supplementary Materials

The following are available online at https://www.mdpi.com/1422-0067/22/6/2951/s1. Figure S1: NES cells and neuronal progeny characterization, Figure S2: Human neurons culture media SEC elution profile, Figure S3: High molecular weight protein standard SEC elution profile, Table S1: Gene Ontology of proteins identified from NES-NDsEV, Table S2: Comparison of the identified proteins from NES-NDsEV with the Human Brain Proteome database, Table S3: Proteins identified from plasma-NDsEV of PD patients (PD-NDsEV) in common with the Top 100 ExoCarta database, Table S4: Proteins identified from plasma-NDsEV of PD patients belonging to the Gene Ontology Cellular component term ‘Early Endosome’, Table S5: Proteins identified from plasma-NDsEV of PD patients belonging to the Gene Ontology Cellular Component term ‘Endosome’, Table S6: Gene Ontology of the proteins identified from plasma-NDsEV of PD patients, Table S7: Gene Ontology of the proteins identified from plasma-NDsEV of PD patients, Dataset SD1: Identified protein groups from cultured human neurons derived small extracellular vesicles (NES-NDsEV), Dataset SD2: Identified protein groups from PD patients’ plasma-NDsEV, Dataset SD3: Identified proteins that are in common to the 4 patients plasma-NDsEV, Dataset SD4: Identified proteins that are in common between NES-NDsEV and the total plasma-NDsEV proteins, Dataset SD5: Identified proteins that are in common to NES-NDsEV and shared by all the four PD patients plasma-NDsEV.

## Abbreviations

sEV	small extracellular vesicles
NDsEV	neuron-derived small extracellular vesicles
NES	neuro-epithelial stem cells
NES-NDsEV	small extracellular vesicles derived from neuron differentiated from NES cells
PD-NDsEV	neuron-derived small extracellular vesicles isolated from Parkinson Disease patients’ plasma
CNS	central nervous system
LC-MS/MS	liquid chromatography–tandem mass spectrometry
LB	lysis buffer
SDS	sodium dodecyl sulfate
TEAB	triethylammonium bicarbonate
DTT	dithiothreitol
IAA	iodoacetamide
PBS	phosphate buffered saline
FDR	false discovery rate
GO	gene ontology
RT	room temperature.
