# Supplementary material for: Proteomics Profiling of Neuron-Derived Small Extracellular Vesicles from Human Plasma: Enabling Single-Subject Analysis
Source: Int J Mol Sci. 2021 Mar 14;22(6):2951. doi: 10.3390/ijms22062951 (PMC7999506; doi:10.3390/ijms22062951)
Supplement: Supplementary file 1 [file ijms-22-02951-s001.zip › Supplementary Material/Supp_Info_IJMS_NDsEV_Proof_Confirm.pdf]

Supplementary Information

Proteomics profiling of neuron-derived small extracellular vesicles from human plasma: enabling single subject analysis

Federica Anastasi [1,2], Silvia Maria Masciandaro [3], Renata Del Carratore [3,*], Maria Teresa Dell'Anno [2], Giovanni Signore [2], Alessandra Falleni [4], Liam A. McDonnell [2,*] and Paolo Bongioanni [5,6]

[1] National Enterprise for nanoScience and nanoTechnology, Scuola Normale Superiore, Pisa 56127, Italy

[2] Fondazione Pisana per la Scienza ONLUS, San Giuliano Terme (PI) 56107, Italy

[3] National Research Council, Institute of Clinical Physiology Research, Pisa 56124, Italy

[4] Department of Clinical and Experimental Medicine, Unit of Applied Biology and Genetics, University of Pisa, Pisa 56126, Italy

[5] Severe Acquired Brain Injuries Dpt Section, Azienda Ospedaliero-Universitaria Pisana, Pisa 56126, Italy

[6] NeuroCare onlus, Pisa 56121, Italy

AUTHOR INFORMATIONS

*Corresponding Authors:

Renata Del Carratore Email: rdc@ifc.cnr.it

Liam A. McDonnell Email: liam@fpscience.it

Supplementary Figures

Figure S1. NES cells and neuronal progeny characterization. (A-C) NES cells are immunopositive for neural stem cell markers Nestin and Vimentin (VIM), and express transcription factors SOX1 and SOX2, typically found in neuronal progenitors. Phospho-vimentin (pVIM) labels cells in the M phase of the cell cycle. (D-F) Upon three months of differentiation, NES cell-derived neurons appear immunodecorated with pan-neuronal markers neurofilament (NF), MAP2, and TUBB3, mostly expressed in neurites. Neuronal somas are immunopositive for RBFOX3. Scale bar is 20 µ~~u~~m.

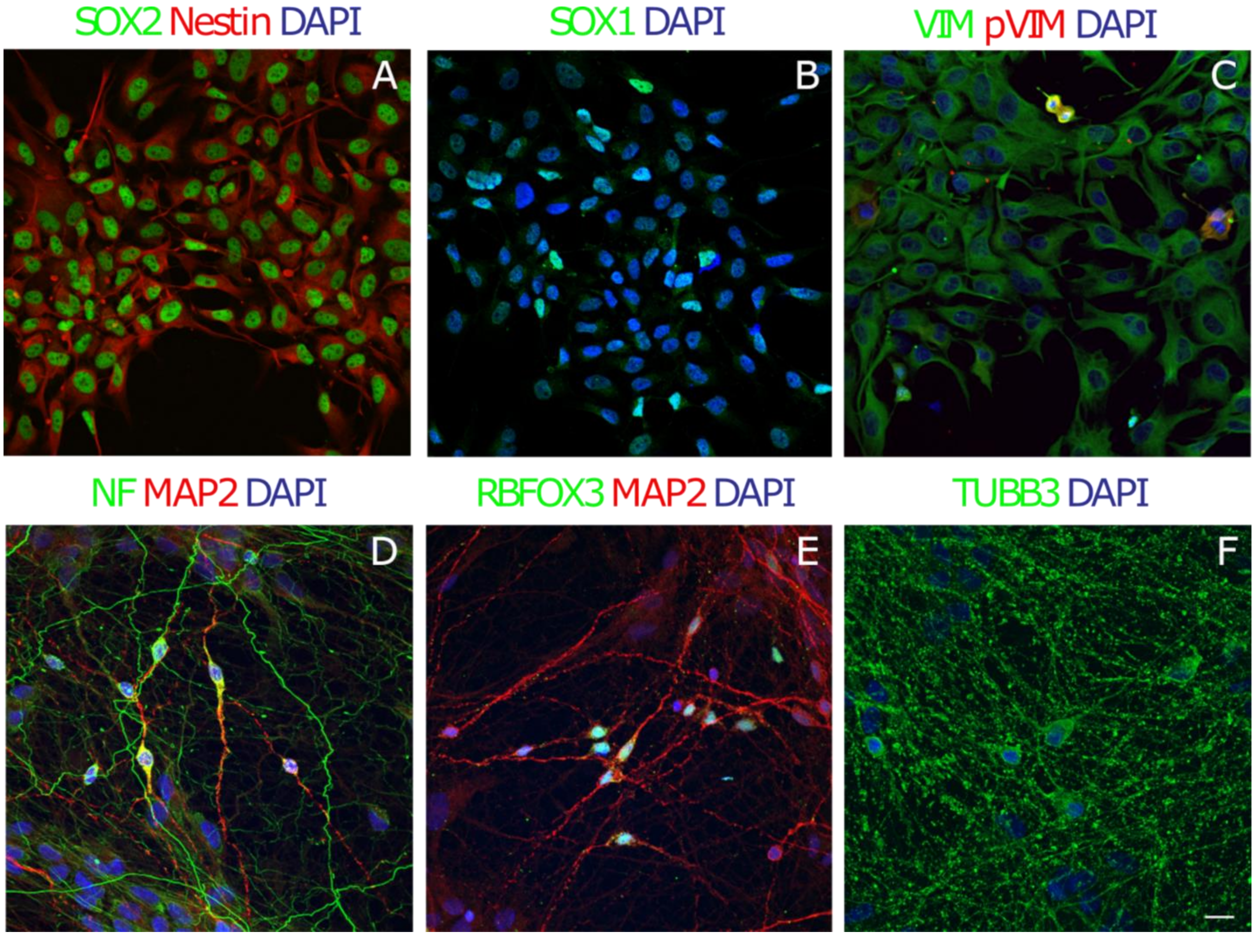

Figure S2. **Human neurons' culture media SEC elution profile.** The analysis was performed using a Superose 6 Increase 10/300 SEC column in-line with a Zetasizer Dynamic Light Scattering. The graph represents the vesicles diameter size (d.nm) in blue, the derived count rate in red and the signal detected at λ 220 nm in black. The light-orange box represents the selected fraction for NDsEV analysis (14-22 min).

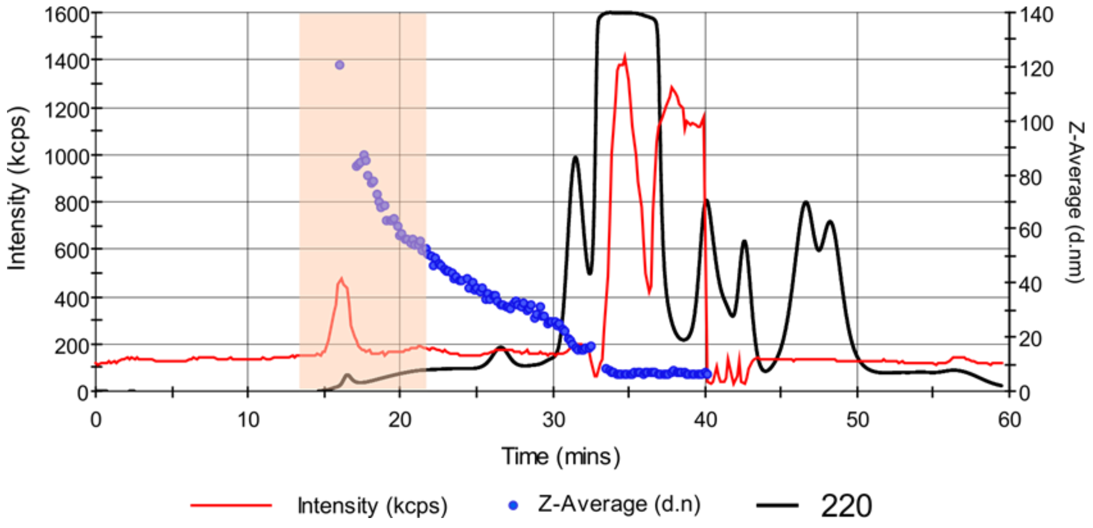

Figure S3. High molecular weight protein standard SEC elution profile. Thyroglobulin (660 kDa), Bovine Serum Albumin (66.5 kDa) and Myoglobin (16.7 kDa) obtained with a Superose 6 Increase 10/300 column at a flow of 0.5 ml/min.

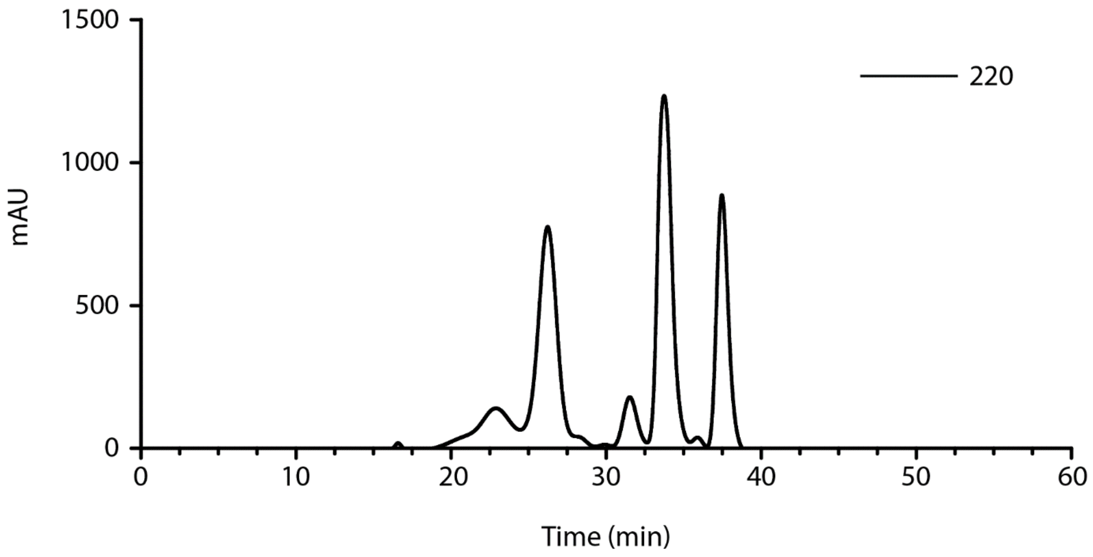

Supplementary Tables

Table S1. Gene Ontology of proteins identified from NES-NDsEV. The analysis was performed with DAVID database using the total *homo sapiens* dataset as reference database. Proteins used for the analysis: 431. Significantly enriched 'Cellular component' GO terms are reported in the table with the relative count number and *p*-value FDR corrected.

**CELLULAR COMPONENT**		
**Term**	**Count**	**FDR**
Extracellular Exosome	311	$1,4 \times 10^{-161}$
Extracellular Space	146	$2,5 \times 10^{-58}$
Blood Microparticle	59	$1,2 \times 10^{-53}$
Extracellular Region	150	$8,2 \times 10^{-52}$
Extracellular Matrix	64	$5,2 \times 10^{-41}$
Focal Adhesion	61	$1,3 \times 10^{-30}$
Extracellular Vesicle	22	$4,0 \times 10^{-20}$
Melanosome	28	$5,3 \times 10^{-20}$
Plasma Membrane	168	$2,2 \times 10^{-14}$
Cell-Cell Adherens Junction	38	$2,4 \times 10^{-14}$
Basement Membrane	21	$2,4 \times 10^{-14}$
Platelet Alpha Granule Lumen	9	3.40×10^{-03}
Vesicle	5	8.60×10^{-03}
Myelin Sheath	4	1.60×10^{-02}
Cell Surface	6	3.30×10^{-02}

Table S2. Comparison of the identified proteins from NES-NDsEV with the Human Brain Proteome database (Human Proteome Atlas, https://www.proteinatlas.org/humanproteome/brain/human+brain). The table reports the proteins identified in common between the NES-NDsEV and the proteins annotated as elevated in the brain (2582 entries).

**Gene**	**Protein description**
ARF3	ADP ribosylation factor 3
SLC44A1	solute carrier family 44 member 1
NCAN	neurocan
DIRAS2	DIRAS family GTPase 2
DBH	dopamine beta-hydroxylase
CELSR2	cadherin EGF LAG seven-pass G-type receptor 2
BASP1	brain abundant membrane attached signal protein 1
C1QTNF5	C1q and tumor necrosis factor related protein 5
ENPP2	ectonucleotide pyrophosphatase/phosphodiesterase 2
RAC3	ras-related C3 botulinum toxin substrate 3 (rho family, GTP binding protein Rac3)
NRCAM	neuronal cell adhesion molecule
EDIL3	EGF like repeats and discoidin domains 3
CHST6	carbohydrate sulfotransferase 6
METRN	meteorin, glial cell differentiation regulator
GPR37L1	G protein-coupled receptor 37 like 1
GABRG3	gamma-aminobutyric acid type A receptor gamma3 subunit
F5	coagulation factor V
BCAN	brevican
GAP43	growth associated protein 43
GPRC5B	G protein-coupled receptor class C group 5 member B
RAP2A	RAP2A, member of RAS oncogene family
CRISPLD1	cysteine rich secretory protein LCCL domain containing 1
COL4A5	collagen type IV alpha 5 chain
ALDOC	aldolase, fructose-bisphosphate C
TNIK	TRAF2 and NCK interacting kinase
LY6H	lymphocyte antigen 6 complex, locus H
PFN2	profilin 2
IGSF8	immunoglobulin superfamily member 8
GPM6A	glycoprotein M6A
TTYH2	tweety family member 2
CNP	2',3'-cyclic nucleotide 3' phosphodiesterase
TTYH1	tweety family member 1
LYPD1	LY6/PLAUR domain containing 1

SLC1A3	solute carrier family 1 member 3
CRMP1	collapsin response mediator protein 1
THY1	Thy-1 cell surface antigen
FSTL5	follistatin like 5
PTPRZ1	protein tyrosine phosphatase, receptor type Z1
TSPAN9	tetraspanin 9
MVB12B	multivesicular body subunit 12B
SPOCK1	SPARC/osteonectin, cwcv and kazal like domains proteoglycan 1
NCAM1	neural cell adhesion molecule 1 or L1CAM
CKB	creatine kinase B
GPM6B	glycoprotein M6B
BTBD17	BTB domain containing 17
BDNF	brain derived neurotrophic factor
ST8SIA1	ST8 alpha-N-acetyl-neuraminide alpha-2,8-sialyltransferase 1
PCDHGC3	protocadherin gamma subfamily C, 3
LSAMP	limbic system-associated membrane protein
HSPA2	heat shock protein family A (Hsp70) member 2
SMOC1	SPARC related modular calcium binding 1
PLP1	proteolipid protein 1
CSPG5	chondroitin sulfate proteoglycan 5
HRG	histidine rich glycoprotein
FOLR1	folate receptor 1
CAPS	calcyphosine
ITM2C	integral membrane protein 2C

Table S3. Proteins identified from plasma-NDsEV of PD patients in common with the Top100 ExoCarta database. The table reports the proteins shared between the ExoCarta Top 100 database (www.exocarta.org) and the plasma-NDsEV with relative replicate's number of samples in which they have been identified.

**Gene name**	**Description**	**# replicates**
ANXA2	Annexin A2	6
A2M	Alpha-2-macroglobulin	6
GAPDH	Glyceraldehyde-3-phosphate dehydrogenase	6
ALDOA	Fructose-bisphosphate aldolase	6
TPI1	Triosephosphate isomerase	5
HSPA5	Endoplasmic reticulum chaperone BiP	5
PRDX1	Peroxiredoxin-1	5
HSPA8	Heat shock cognate 71 kDa protein	5
PRDX2	Peroxiredoxin-2	5
ENO1	Alpha-enolase	5
PKM	Erythrocyte band 7 integral membrane protein	5
ANXA1	Annexin A1	4
AHCY	Adenosylhomocysteinase	4
STOM	Erythrocyte band 7 integral membrane protein	4
LGALS3BP	Galectin-3-binding protein	4
EEF1A1	Elongation factor 1-alpha 1	4
EEF2	Elongation factor 2	3
GNAI2	Guanine nucleotide-binding protein G(i) subunit alpha-2	3
FLOT1	Flotillin-1	3
TKT	Transketolase	2
CFL1	Cofilin-1	2
MYH9	Myosin-9	2
LDHA	Flotillin-1 OS=Homo sapiens	2
PFN1	Profilin-1	2
CDC42	Cell division control protein 42 homolog	2
CD9	CD9 antigen	2
TFRC	Transferrin receptor protein 1	2
FLNA	Filamin-A	2
PGK1	Phosphoglycerate kinase 1	2
MSN	Moesin	1
MFGE8	Lactadherin	1
THBS1	Thrombospondin-1	1
GDI2	Rab GDP dissociation inhibitor bet	1
YWHAZ	14-3-3 protein zeta/delta OS=Homo sapien	1
ITGB1	Erythrocyte band 7 integral membrane protein	1

RAB1A	Ras-related protein Rab-1A	1
VCP	Transitional endoplasmic reticulum ATPase	1
RAN	GTP-binding nuclear protein Ran	1
RAB7A	Ras-related protein Rab-7a	1
YWHAB	14-3-3 protein beta/alpha	1
ACTN4	Alpha-actinin-4	1

Table S4. Gene Ontology of the proteins identified from plasma-NDsEV of PD patients. The analysis was performed with DAVID database using the total *homo sapiens* dataset as reference database. The analysis was performed on the 177 protein groups in common to all the 4 PD patients' samples. Significantly enriched 'Cellular component' GO terms are reported in the table with the relative count number and *p*-value FDR corrected.

**CELLULAR COMPONENT**		
**Term**	**Count**	**FDR**
Blood microparticle	74	$1,5 \times 10^{-112}$
Extracellular region	127	$1,9 \times 10^{-99}$
Extracellular exosome	140	$7,9 \times 10^{-88}$
Extracellular space	110	$4,6 \times 10^{-83}$
Platelet alpha granule lumen	22	$3,7 \times 10^{-28}$
High-density lipoprotein particle	13	$2,2 \times 10^{-18}$
Extracellular matrix	26	$2,4 \times 10^{-16}$
Very-low-density lipoprotein particle	11	$6,9 \times 10^{-15}$
Membrane attack complex	7	$4,9 \times 10^{-11}$
Spherical high-density lipoprotein particle	7	$1,6 \times 10^{-10}$
Chylomicron	8	$1,6 \times 10^{-10}$
Endocytic vesicle lumen	8	$4,9 \times 10^{-10}$
Platelet dense granule lumen	7	$1,4 \times 10^{-08}$
Fibrinogen complex	6	$6,4 \times 10^{-08}$
External side of plasma membrane	15	$6,4 \times 10^{-08}$

Table S5. Gene Ontology of the proteins identified from plasma-NDsEV of PD patients. The analysis was performed with DAVID database using the total *homo sapiens* dataset as reference database. The analysis was performed on the total number of identified proteins (630). Significantly enriched 'Cellular component' GO terms are reported in the table with the relative count number and *p*-value FDR corrected.

**CELLULAR COMPONENT**		
**Term**	**Count**	**FDR**
Extracellular Exosome	411	$1{,}2 \times 10^{-198}$
Blood Microparticle	102	$1{,}8 \times 10^{-112}$
Extracellular Region	251	$4{,}0 \times 10^{-109}$
Extracellular Space	230	$6{,}6 \times 10^{-107}$
Platelet Alpha Granule Lumen	37	$1{,}0 \times 10^{-38}$
Extracellular Matrix	67	$7{,}8 \times 10^{-35}$
High-Density Lipoprotein Particle	16	$3{,}2 \times 10^{-16}$
Focal Adhesion	49	$8{,}8 \times 10^{-14}$
Very-Low-Density Lipoprotein Particle	14	$1{,}1 \times 10^{-13}$
Cell-Cell Adherens Junction	43	$6{,}0 \times 10^{-13}$
Cytosol	182	$1{,}6 \times 10^{-12}$
Melanosome	24	$3{,}8 \times 10^{-12}$
Platelet Dense Granule Lumen	11	$3{,}3 \times 10^{-11}$
Chylomicron	11	$3{,}3 \times 10^{-11}$
External Side Of Plasma Membrane	32	$6{,}3 \times 10^{-11}$

Table S6. Proteins identified from plasma-NDsEV of PD patients belonging to the Gene Ontology Cellular component term 'Early Endosome'.

**Early Endosome (GO:0005769)**	
**Gene name**	**Description**
ANXA2	Annexin A2
APOB	Apolipoprotein B-100
APOA1	Apolipoprotein A-I
APOA5	Apolipoprotein A-V
APOC2	Apolipoprotein C-II
APOA4	Apolipoprotein A-IV
HSPD1	60 kDa heat shock protein, mitochondrial
RAB1A	Ras-related protein Rab-1A
APOE	Apolipoprotein E
EEA1	Early endosome antigen 1
PLA2G4E	Cytosolic phospholipase A2 epsilon
TFRC	Transferrin receptor protein 1

FLOT1	Flotillin-1
LTF	Lactotransferrin
STX7	Syntaxin-7
APOA2	Apolipoprotein A-II
TF	Serotransferrin
B2M	Beta-2-microglobulin
APOC3	Apolipoprotein C-III
ANXA1	Annexin A1

Table S7. Proteins identified from plasma-NDsEV of PD patients belonging to the Gene Ontology Cellular Component term 'Endosome'.

**Endosome (GO:0005768)**	
**Gene name**	**Description**
RAN	GTP-binding nuclear protein Ran
HLA-DRB1	HLA class II histocompatibility antigen, DRB1-15 beta chain
STEAP3	Metalloreductase STEAP3
ANXA2	Annexin A2
CD14	Monocyte differentiation antigen CD14
HSPA8	Heat shock cognate 71 kDa protein
APOB	Apolipoprotein B-100
APOA1	Apolipoprotein A-I
CDH1	Cadherin-1
RAB11B	Ras-related protein Rab-11B
FLOT2	Flotillin-2
ATP6V0A1	V-type proton ATPase 116 kDa subunit a isoform 1
APOA5	Apolipoprotein A-V
CTSD	Cathepsin D
IFITM3	Interferon-induced transmembrane protein 3
ANTXR1	Anthrax toxin receptor 1
APOC2	Apolipoprotein C-II
APOA4	Apolipoprotein A-IV
HSPD1	60 kDa heat shock protein, mitochondrial
RAB1A	Ras-related protein Rab-1A
APOE	Apolipoprotein E
TPP1	Tripeptidyl-peptidase 1
GRN	Progranulin
EEA1	Early endosome antigen 1
HLA-DRA	HLA class II histocompatibility antigen, DR alpha chain
PLA2G4E	Cytosolic phospholipase A2 epsilon
TFRC	Transferrin receptor protein 1

LAMP1	Lysosome-associated membrane glycoprotein 1
RPS27A	Ubiquitin-40S ribosomal protein S27a
RAB7A	Ras-related protein Rab-7a
FLOT1	Flotillin-1
LTF	Lactotransferrin
HRG	Histidine-rich glycoprotein
TMBIM1	Protein lifeguard 3
STX7	Syntaxin-7
APOA2	Apolipoprotein A-II
TF	Serotransferrin
B2M	Beta-2-microglobulin
ITGB1	Integrin beta-1
PDCD6	Programmed cell death protein 6
APOC3	Apolipoprotein C-III
ANXA1	Annexin A1
RAN	GTP-binding nuclear protein Ran
HLA-DRB1	HLA class II histocompatibility antigen, DRB1-15 beta chain
